# The Phenolic Composition of *Centaurea cyanus* L. Flower Extracts: Antioxidant, Antimicrobial, and Fibroblast Biocompatibility of a Phenolic-Rich Ethanolic Extract

**DOI:** 10.3390/ijms27188101

**Published:** 2026-09-11

**Authors:** Urszula Kalinowska-Lis, Lena Czerwonka, Ewelina Piątczak, Weronika Gonciarz, Joanna Kolniak-Ostek

**Affiliations:** 1 Department of the Chemistry of Cosmetic Raw Materials, Faculty of Pharmacy, Medical University of Lodz, Muszyńskiego 1, 90-151 Lodz, Poland; lena.czerwonka@student.umed.lodz.pl; 2 Department of Pharmaceutical Biotechnology, Faculty of Pharmacy, Medical University of Lodz, Muszyńskiego 1, 90-151 Lodz, Poland; ewelina.piatczak@umed.lodz.pl; 3 Department of Immunology and Infectious Biology, Faculty of Biology and Environmental Protections, University of Lodz, Banacha 12/16, 90-237 Lodz, Poland; weronika.gonciarz@biol.uni.lodz.pl; 4 Department of Fruit, Vegetable and Plant Nutraceutical Technology, Wrocław University of Environmental and Life Sciences, Chełmońskiego 37, 51-630 Wroclaw, Poland; joanna.kolniak-ostek@upwr.edu.pl

**Keywords:** antioxidative activity, antimicrobial, apigenin, *Centaurea cyanus* L., extracts, fibroblasts, medicinal plant, phytochemistry, spermidine

## Abstract

Six *Centaurea cyanus* L. flower extract variants were prepared using methanol, ethanol or water as a solvent; in each case, the material was either defatted with chloroform or not. The phytochemical profile of all obtained extracts was analyzed qualitatively and quantitatively using UPLC-PDA-Q/TOF-MS. In total, 35 phenolic compounds were detected, including flavonols, flavones, anthocyanins, phenolic acids and their derivatives, as well as an N-*p*-coumaroyl spermidine derivative; some compounds were identified using authentic standards, while others were tentatively identified based on chromatography and mass spectrometry data. The defatted ethanolic extract showed the highest total quantified phenolic content (42.18 mg/g DW), with the predominant constituents being apigenin derivatives, particularly apigenin-*O*-hexoside-*O*-glucuronide and apigenin-3-*O*-glucoside-(6″-acetyl)glucoside. The extract exhibited notably high antioxidant capacity, viz. DPPH (240.34 µmol TE/g), ABTS (170.53 µmol TE/g), and FRAP (189.93 µmol TE/g), and moderate antimicrobial activity, particularly against *E. coli* (MIC 0.5 mg/mL). The extract did not exhibit any cytotoxic effect toward L929 or Hs68 fibroblasts in vitro at concentrations up to 5 mg/mL. These findings indicate that the phenolic-rich ethanolic extract demonstrated both antioxidant and antimicrobial activities while also being non-cytotoxic against fibroblasts within the tested concentration range; as such, it may be suitable for dermatological and cosmetic formulations.

## 1. Introduction

Recent years have seen growing interest in the potential applications of plant raw materials in medicine, pharmacology and cosmetology. One such plant is cornflower (*Centaurea cyanus* L.–Asteraceae). This species occurs naturally in Europe and Western Asia, where it is considered a segetal weed due to its frequent co-occurrence in cereal crops, particularly rye, wheat, and barley. While the intensification of agriculture and herbicide use have reduced its populations in Western Europe, the species is still relatively common in Central and Eastern Europe, especially on light and sandy soils [1,2].

The plant is an annual, characterized by an erect, hairy stem (20–90 cm in height), branching at the top and ending in capitulum-type inflorescences with a diameter of 1.5–3 cm. The inflorescences consist of bunches of violet tubular flowers located in the central part of the capitulum, with intense blue, ligulate flowers arranged around its margin. The leaves are alternately arranged, lanceolate, with entire margins and narrowing toward the base; the lower leaves are sometimes pinnately divided [1,3].

Thanks to their antipruritic, antitussive, astringent, emmenagogue and mild laxative properties, preparations obtained from *Centaurea cyanus* L. have a long history of use in traditional medicine, where they have also been applied to relieve ocular fatigue [3,4]. Furthermore, extracts from a number of *Centaurea* L. species have been found to demonstrate beneficial anti-inflammatory, cytotoxic, antidiabetic, antidepressant, antioxidant, antimicrobial and gastroprotective properties [5,6,7,8,9,10,11,12,13].

*Centaurea cyanus* L. is widely used in cosmetic products, such as toners, hydrolates, and cleansing masks, due to its anti-inflammatory, soothing and antioxidant properties [5,14]. Its active substances are believed to support the natural regenerative mechanisms of the skin, improving its elasticity, protecting it from oxidative stress, and slowing the ageing process. They also exhibit anti-inflammatory effects, strengthen blood vessels, and protect against UV radiation. Due to its mild action, cornflower is considered a safe ingredient and is well tolerated, even by people with sensitive skin [15,16].

Its biological properties have been attributed to the presence of numerous secondary metabolites, including flavonoids such as quercetin, kaempferol, isorhamnetin, apigenin and luteolin derivatives, as well as inter alia anthocyanidins, phenolic acids (caffeic, chlorogenic, isochlorogenic, ferulic, p-coumaric, salicylic, protocatechuic and syringic), polysaccharides, coumarins, tocopherols, terpenes and carotenoids [3,5,6,9,11].

Although previous studies have examined the phytochemical composition and selected biological properties of *Centaurea cyanus* flowers, little is known about the influence of extraction conditions on the recovery of their phenolic constituents and extract composition. In particular, few attempts have been made to make systematic comparisons between the effectiveness of different extraction solvents with an initial defatting step. Moreover, few studies have taken a more comprehensive approach by comparing the phenolic profiles of differently prepared plant extracts, evaluating their antioxidant and antimicrobial profiles, and determining their cytocompatibility with fibroblasts.

The initial objective of the study was to determine the influence of the type of extraction solvent and sample preparation procedure on the qualitative and quantitative phenolic composition in cornflower (*Centaurea cyanus* L.) flower extracts. To achieve this, three pairs of extracts were created using different solvents (viz. methanolic, ethanolic or aqueous); each pair comprised one extract that was defatted with chloroform and the other that was not, making a total of six extracts.

Another aim was to identify the extract with the most chemically diverse and quantitatively rich phenolic profile; this variant was then taken for further evaluation of its antioxidative effects, its antimicrobial properties against various human pathogens, including skin pathogens, and its cytotoxic activity against human and murine skin fibroblasts. The obtained data regarding its biological activity can be used to confirm its suitability as an active component in cosmetic formulations intended for sensitive, acne-prone, or ageing skin; such formulations can be used for regenerating or protecting skin, or mitigating and calming lesions. In addition, the in vitro cytotoxicity data can be used to estimate the optimal concentration of the extract in a given cosmetic formulation so that it does not have a toxic effect on skin cells.

The systematic comparison of six extraction variants based on different solvent types and the optional use of a preliminary defatting step extends previous research on the pharmacological value of *C. cyanus* flowers. Also, by integrating a detailed analysis combining the phenolic profile, antioxidant and antimicrobial activities and fibroblast cytocompatibility of *C. cyanus* flower extracts, it offers a comprehensive assessment of the influence of extraction strategy on their chemical composition and biological potential.

## 2. Results and Discussion

### 2.1. Qualitative Analysis of Phenolic Compounds

The six extracts obtained from *Centaurea cyanus* flowers were found to contain a diverse profile of phenolic compounds (Table 1). A total of 35 individual phenolics were identified, comprising flavonols, flavones, phenolic acids, anthocyanins, and N-*p*-coumaroyl spermidine derivatives.

The predominant flavonols in the extracts included various kaempferol and quercetin derivatives, as well as single derivatives of isorhamnetin, axillarin and taxifolin. Peaks **19**–**22** revealed pseudomolecular ions [M−H]^−^ at *m*/*z* 463, 505, 463 and 609; these were tentatively identified as quercetin glucoside, quercetin-3-*O*-(6″-acetyl)glucoside, quercetin galactoside and quercetin hexoside rhamnoside, respectively. Each of these glycosides, after fragmentation, gave a peak typical of a quercetin residue with a mass of 301 u. Quercetin derivatives have already been identified in other flower extracts, including *R. canina*, *C. officinalis* and *C. cyanus* [17,18]. Although less abundant than apigenins, quercetin derivatives demonstrate exceptional antioxidant potential. Previous research has identified a broad spectrum of quercetin, luteolin, and apigenin glycosides in *C. cyanus* flower heads, which demonstrate high reducing power and strong radical-scavenging activities [13].

Among the identified dihydroquercetin derivatives were taxifolin dihexoside isomers (peaks **6** and **7**). Further flavonol compounds, represented by peaks **11**, **14**, **23** and **25**, were annotated as kaempferol derivatives. Of these, peaks **11**, **14**, and **23**, represented by identical molecular ions ([M-H]^−^ at *m*/*z* 447), were assigned to kaempferol hexosides (glucoside or galactoside) following fragmentation of the hexoside moiety (−162 u). Compound **25** [M-H]^−^ at *m*/*z* 489 was identified as kaempferol-*O*-acetylhexoside, which gave free kaempferol (285 u) after fragmentation of the acetylhexoside residue (−204 u). Another *O*-methylated flavonol derivative was axillarin hexoside (peak **17**) ([M-H]^−^ at *m*/*z* 507). This was not present in any previously analyzed cornflower extracts. In addition, isorhamnetin glucuronide was identified (peak **27**); the presence of its fragmentation ion (*m*/*z* 315) can be assigned to the loss of a glucuronide residue (−176 u) from the main ion (*m*/*z* 491). However, this compound was detected in significant amounts only in the two methanol extracts and a non-defatted ethanol extract.

Flavones were represented by free apigenin ([M-H]^−^ at *m*/*z* 269) (peak **30**) and four apigenin glycosides: apigenin-*O*-hexoside-*O*-glucuronide (peak **15**), apigenin-*O*-glucuronide-*O*-(malonylhexoside) (peak **16**), apigenin-3-*O*-glucoside-(6″-acetyl)glucoside (peak **24**) and apigenin-*O*-acetylhexoside (peak **26**), with characteristic molecular ions at *m*/*z* 607, 693, 641 and 473, respectively. Fragmentation ions were identified at *m*/*z* 445, 431 and 269; these were formed by the loss of hexosyl (−162 u), glucuronyl (−176 u) or malonylhexosyl (−248 u) moieties from apigenin derivatives **15** ([M-H]^−^ at *m*/*z* 607) and **16** ([M-H]^−^ at *m*/*z* 693). Molecular ions at *m*/*z* 641 and 473 and the corresponding fragment ions revealed the structures of the compounds **24** and **26**, for which loss of the acetylhexosyl group (−204 u) was observed. This is in line with previous reports indicating apigenin-type flavones as characteristic metabolites of *C. cyanus* flowers. Lockowandt et al. demonstrated that apigenin glucuronides are central components of protocyanin, the supramolecular blue pigment complex typical of the species [18]. Similarly, Popowska and Oracz identified substantial levels of apigenin-7-*O*-glucuronide in both blue and pink cornflower cultivars. Hence, apigenin conjugates appear to form a chemotaxonomic hallmark of *C. cyanus* flowers [19].

A single flavone derivative was identified: eriodictyol hexoside (peak **2**). This has previously been observed in a hydromethanolic extract of dahlia (*Dahlia mignon*) petals [17], and in the inedible lower part of the cornflower capitula [18]. Peak **2** ([M-H]^−^ at *m*/*z* 449) yielded a fragmentation ion at *m*/*z* 287 ([M-H-162]^−^).

Among the phenolic acids, derivatives of caffeic acid and p-coumaric acid (*p*-hydroxycinnamonic acid) predominated. The caffeic acid derivatives included two caffeoylhexose (peaks **3** and **4**), two 3-caffeoylquinic acids (peaks **8** and **9**) and acetyl-caffeoyl quinic acid (peak **28**). Peaks **8** and **9** presented molecular ions [M-H]ˉ at *m*/*z* 353 and ions at *m*/*z* 191 (quinic acid) that were associated with the loss of a caffeoyl moiety (−162 u). Derivatives of *p*-coumaric acids included two *p*-coumaric acid hexosides (peaks **10** and **12**) and two *p*-coumaroylquinic acids (peaks **13** and **18**). The predominance of caffeoylquinic acid derivatives, including 3-caffeoylquinic acid, may partially explain the biological activity of the extracts. Chlorogenic acid has been widely reported to possess antioxidant activity and has also demonstrated anticancer potential through the induction of apoptosis in cancer cell models [20,21].

Among the anthocyanins, five cyanidin derivatives were identified. Peak **31** (*m*/*z* 611) was identified as cyanidin-3,5-*O*-diglucoside; this was associated with fragmentation ions at *m*/*z* 449 and 287 formed by the loss of two glucoside residues (−162 u). This derivative was previously described by Escher et al. [9] and Lockowandt et al. [18]. Peaks **32** and **33** ([M+H]^+^ at *m*/*z* 697 and 711) were identified as cyanidin-3,5-*O*-(6″-malonylglucoside)-*O*-glucoside and cyanidin-3,5-*O*-(6″-succinylglucoside)-*O*-glucoside, respectively [9,18]. Peak **35** ([M+H]^−^ at *m*/*z* 535) was identified as cyanidin-3-*O*-(6-*O*-malonylglucoside); the compound is almost identical to compound 32, but lacking a glucoside group. Finally, compound **34** (*m*/*z* 625) was described as cyanidin-3-O-glucoside monoglucuronide. It formed a fragmentation ion at *m*/*z* 287 following the loss of part of the glucuronide moiety (−176 u) and the glucoside group (−162 u). Anthocyanins were described as characteristic cyanidin conjugates by Lockowandt et al. [18] and Popowska and Oracz [19], and despite their lower abundance, they may play a structural or stabilizing role within the phenolic matrix.

Peak **29** ([M-H]^−^ at *m*/*z* 582) was identified as N^1^,N^5^,N^10^-tri-*p*-(*E,E,E*)-coumaroyl spermidine, with characteristic fragmentation ions at *m/z* 462, 342, 145 and 119. This fragmentation pattern was similar to that of N^1^,N^5^,N^10^-tri-*p*-(*E,E,E*)-coumaroyl spermidine, found to be present in other plant families [22,23]. N^1^,N^5^,N^10^-tri-*p*-(*E*,*E*,*E*)-coumaroyl spermidine has previously been reported in *C. cyanus* capitula, particularly in the non-edible lower part of the flower heads [18]. In the present study, this compound was also detected in flower extracts prepared with different solvents. Its detection supports earlier observations highlighting the presence of hydroxycinnamoyl polyamines in the reproductive organs of the Asteraceae. These compounds exhibit moderate antioxidant potential and may contribute to the plant’s defensive response [24,25].

### 2.2. Quantitative Analysis of Centaurea cyanus L. Extracts

**Figure 1:**
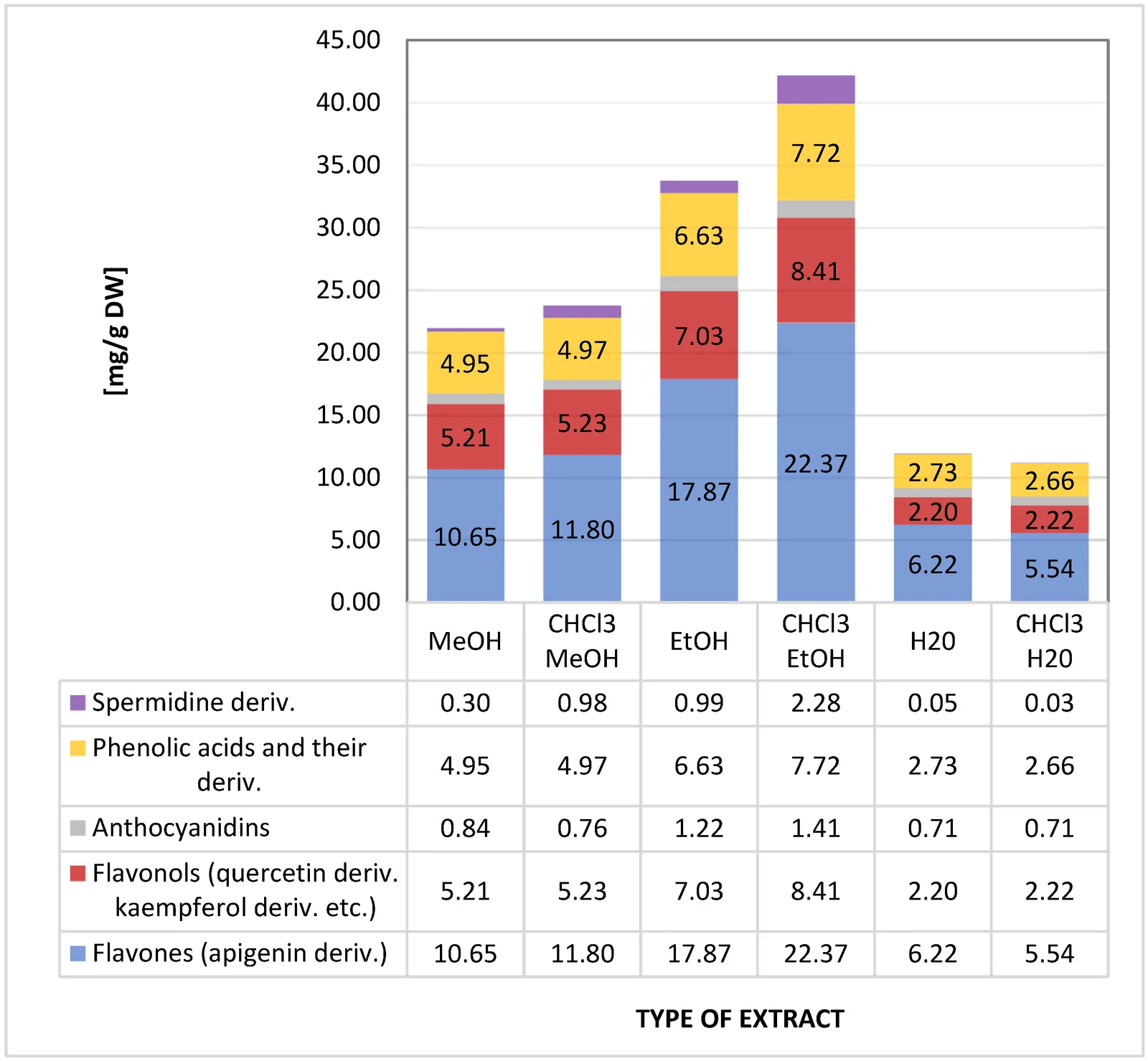
The quantities of ingredients from individual groups depending on the type of extract.

Six *Centaurea cyanus* L. flower extracts were used in the experiment, depending on the solvent type (ethanolic, methanolic or aqueous solvent) and whether or not the material had been defatted with chloroform. The resulting six variants were marked as follows: methanolic extract, not defatted MeOH (**1**); methanolic extract, defatted CHCl_3_, MeOH (**2**); ethanolic extract, not defatted EtOH (**3**); ethanolic extract, defatted CHCl_3_, EtOH (**4**); aqueous extract, not defatted H_2_O (**5**); and aqueous extract, defatted CHCl_3_, H_2_O (**6**). The quantitative analysis of those extracts (**1–6**) is given in Table 1 and Figure 1.

The ethanolic extracts were the richest in phenolic compounds, followed by the methanolic extracts, and finally the aqueous extracts. Among the two ethanolic extracts, the defatted extract (4) was significantly richer in phenolic compounds. Extract (**4**) also contained approximately twice as many polyphenolic compounds as the methanol extract, and approximately four times more than the aqueous extract.

The highest total phenolic content reached 42.18 mg/g DW, observed in ethanolic extract (**4**). This is consistent with the view that cornflower flowers represent one of the most phenolic-rich floral materials within the *Asteraceae*. This value is similar to that noted in a previous study, i.e., 38.00 mg/g DW [18].

Extract (**4**) was dominated by flavones, i.e., apigenin derivatives, which accounted for more than half of the total phenolic fractions, i.e., 22.37 mg/g DW. Among these, the most abundant were apigenin-*O*-hexoside-*O*-glucuronide (peak **15**), apigenin-3-*O*-glucoside-(6″-acetyl)glucoside (peak **24**) and free apigenin (peak **30**); their respective quantities were 13.63 mg/g DW (32.3%), 5.34 mg/g DW (12.7%) and 2.11 mg/g DW (5.0%), for peaks **15**, **24** and **30** (Table 1).

In addition, 19.9% of the total phenolic content of extract (**4**) consisted of flavonols, with quercetin glucoside (peak **21**), i.e., 3.16 mg/g DW (7.5%), and axillarin hexoside (peak **17**), i.e., 1.84 mg/g DW (4.4%), predominating. Notably, the total content of non-anthocyanin flavonoids, viz. flavones, flavonols and phenolic acids, differed from previous studies, i.e., 10.7 mg/g DW. In contrast, greater levels of apigenin derivatives were detected, i.e., 9.41 mg/g DW [18].

The phenolic acids and their derivatives constituted 18.3% of the total phenolic content, with 3-caffeoylquinic acid (peak **8**) predominating (4.34 mg/g DW; 10.3%). This is considerably higher than the values given in previous studies, viz. 0.134 mg/g DW [18] and 3.02 mg/g DW (sum of isomers) [17]. Interestingly, Escher et al. quantified this compound at 39.0 mg/g DW [9]. The 3-caffeoylquinic acid content in the present extract (4.34 mg/g DW) was also substantially higher than noted previously in the herb *C. cyanus* (0.014 mg/g) [26]. Several *Centaurea* species have demonstrated high levels of mono- and di-caffeoylquinic acids [27], which have exhibited strong antioxidant effects in vitro. It can be seen that considerably higher levels of hydroxycinnamic acids accumulate in floral tissue than in vegetative tissue, and the extraction protocol used in this study (chloroform defatting followed by ethanolic extraction) is highly effective in releasing chlorogenic acid-related compounds.

In addition, 5.4% of the total extract content (2.28 mg/g DW) (peak **29**) was constituted by N^1^,N^5^,N^10^-tri-*p*-(*E,E,E*)-coumaroyl spermidine.

Interestingly, anthocyanins (peaks **31–35**) only represented 3.3% of the total phenolic content (1.41 mg/g DW), i.e., approximately 20 times smaller than noted previously (27.0 mg/g DW) [18].

### 2.3. Antioxidant Capacity of Centaurea cyanus *L*. Ethanolic Extract (4)

The extract exhibited high antioxidant capacity, as measured by DPPH (240.34 µmol TE/g), ABTS (170.53 µmol TE/g), and FRAP (189.93 µmol TE/g) (Table 2). These values are within the upper range of antioxidant activities reported for floral extracts and are consistent with earlier observations that *C. cyanus* flowers possess strong radical-scavenging properties. Fernandes et al. [28] and Chiru et al. [29] likewise found that extracts from dried cornflower flowers exhibit high DPPH, ABTS, and FRAP activities, and that these values strongly correlated with total phenolic content.

The substantial antioxidant capacity demonstrated by the selected ethanolic extract, reflected in the results of the DPPH, ABTS and FRAP assays, can be attributed to its high phenolic compound content, characterized by the presence of hydroxycinnamic acids, quercetin glycosides, apigenin derivatives and anthocyanins. These constituents have all previously been associated with radical-scavenging and reducing properties.

However, as all testing was performed on the whole extracts, it is not possible to confidently identify the synergistic or additive interactions between their individual compounds and their defined combinations. Hence, the observed antioxidant capacity should be regarded as a property of the complete phenolic-rich extract, and the relative contributions and possible interactions of individual constituents require further study.

It should be noted that several phenolic classes are present in distinctly higher amounts than noted in previous studies, particularly when contrasted with herbaceous material [26]; as such, care should be taken when selecting the plant part and extraction strategy for the study. In addition, *C. cyanus* has been found to upregulate phenolic biosynthesis under stress conditions (e.g., herbicide resistance); hence, it is possible that pre-extraction processing steps such as defatting or dehydration may enhance phenolic release or stability [13].

### 2.4. Cytotoxic Activity of Centaurea cyanus *L*. Ethanolic Extract (4)

**Figure 2:**
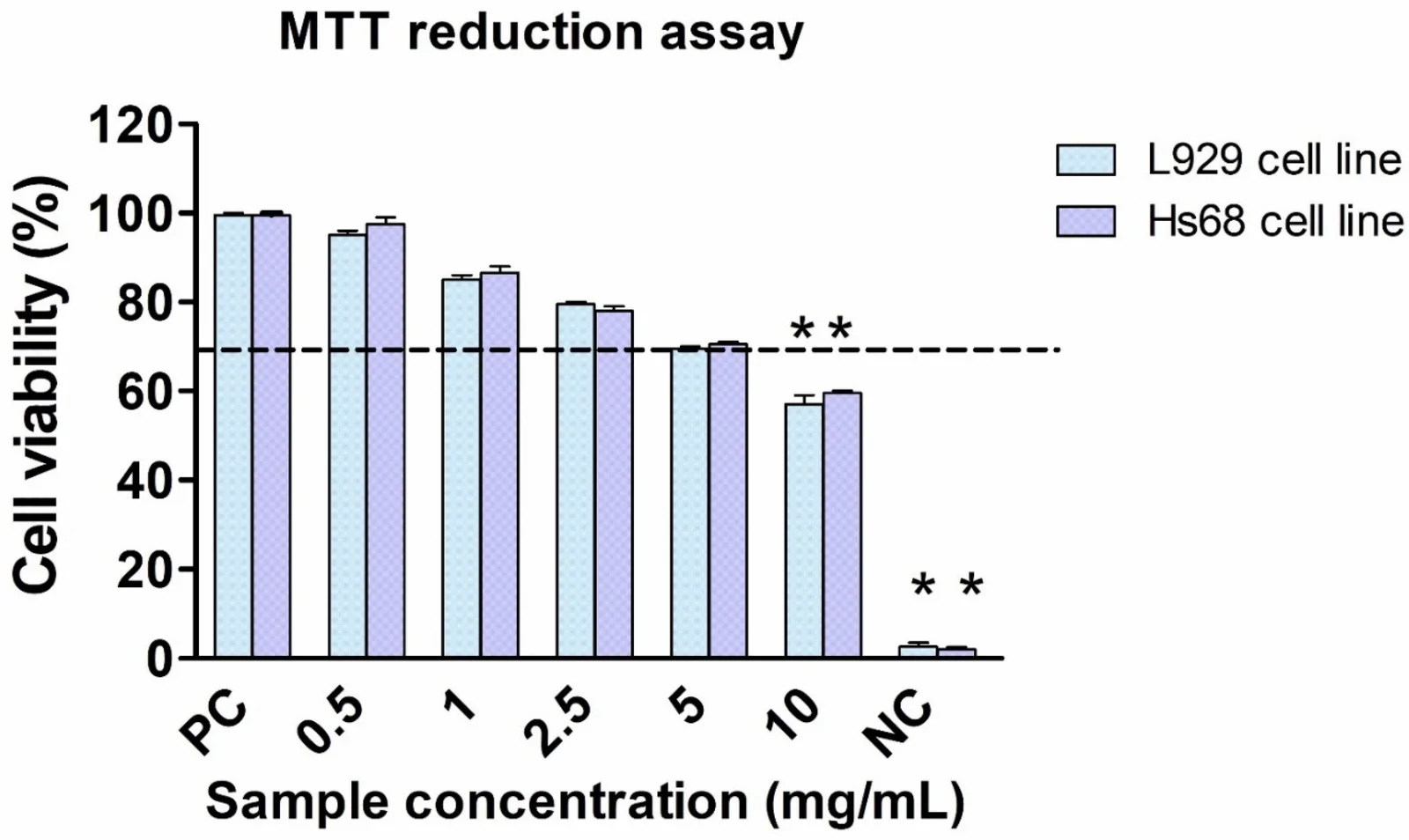
The effect of *Centaurea cyanus* ethanolic extract (**4**) on the viability of mouse L929 fibroblasts and human Hs68 skin fibroblasts was assessed using the MTT reduction assay. The positive viability control (PC; 100% viability) was prepared using cells maintained in complete RPMI-1640 mediumwithout extract, and the negative control (NC) using cells treated with 0.03% H_2_O_2_. The dotted line indicates the 70% cell viability threshold for in vitro cytotoxicity according to ISO 10993-5 [30]. Data are presented as mean ± SD from four independent experiments, each performed in triplicate. Statistical significance was determined separately for each cell line by comparison of untreated cells with cells exposed to the respective extract concentration using the Mann–Whitney U-test; * *p* < 0.05.

The tested extract (4) showed no in vitro cytotoxic effect against reference L929 mouse fibroblasts and human Hs68 skin fibroblasts at concentrations up to 5 mg/mL using the MTT reduction assay. The cells were treated with 0.5–10 mg/mL *C. cyanus* extract (Figure 2). A concentration-dependent cytotoxic effect was noted for both cell lines: viability remained at or above the 70% cytotoxicity threshold up to 5 mg/mL, but fell below 70% at the highest tested concentration (10 mg/mL).

These findings are consistent with previous observations indicating that plant-derived polyphenol-rich extracts may be well tolerated by normal fibroblasts over a relatively broad concentration range [31,32,33]. However, the present results should be interpreted specifically as an assessment of short-term in vitro fibroblast cytocompatibility rather than as evidence of overall biological or dermatological safety.

### 2.5. Antimicrobial Activity of Centaurea cyanus *L*. Ethanolic Extract (4)

The antimicrobial activity of the ethanolic *Centaurea cyanus* flower extract was evaluated against three Gram-negative bacteria (*Pseudomonas aeruginosa*, *Escherichia coli* and *Proteus mirabilis*), four Gram-positive bacteria (*Staphylococcus aureus*, *Staphylococcus epidermidis*, *Bacillus cereus* and *Cutibacterium acnes*) and three fungi (*Candida glabrata*, *Candida albicans* and *Candida parapsilosis*). The extract exhibited moderate antibacterial activity and slightly weaker antifungal activity (Table 3). The MIC and MBC/MFC values were compared with those of gentamicin, amoxicillin and amphotericin B as reference antimicrobials. Among the Gram-positive bacteria, *Staphylococcus epidermidis* was the most susceptible strain, with a MIC of 1 mg/mL and MBC of 2.5 mg/mL. In addition, the extract achieved a MIC of 5 mg/mL and an MBC of 10 mg/mL against *Staphylococcus aureus*, *Bacillus cereus* and *Cutibacterium acnes*.

Among the Gram-negative bacteria, the highest susceptibility was observed for *Escherichia coli*, with an MIC of 0.5 mg/mL and an MBC of 1 mg/mL. This MIC value was lower than that previously reported for hydromethanolic *Centaurea cyanus* flower extracts against *E. coli* (2.5–5.0 mg/mL) [17].

At the evaluated concentrations, the extract exhibited growth inhibition against *Pseudomonas aeruginosa* and *Proteus mirabilis* without a corresponding bactericidal effect; while the MIC was found to be 1 mg/mL, bactericidal activity was still not reached at the highest concentration of 10 mg/mL (MBC > 10 mg/mL).

The extract showed weaker activity against all tested *Candida* spp. While the MIC was achieved at 2.5 mg/mL, MFC was not observed at the highest concentration (MFC > 10 mg/mL; Table 3), indicating that no fungicidal effect was achieved within the reported concentration range. These findings should therefore be interpreted as evidence of limited antifungal activity rather than a strong fungicidal effect.

The antimicrobial activity of *Centaurea* species has been found to vary from low (MIC > 20 mg/mL) to high (MIC < 20 μg/mL) [18,34,35,36,37]. *Centaurea iberica* has also shown antifungal and antimicrobial potential, which has been attributed to its high amounts of sesquiterpene lactones [38]. Methanolic *C. iberica* extract has been found to demonstrate greater antibacterial properties than *C. cyanus* [38]; the extract achieved maximum zones of inhibition of 20 ± 1.5 mm against *K. pneumoniae* (ATCC 1705), 19 ± 1.24 mm against *S. aureus* (ATCC 6538) and 14 ± 1.16 mm against *E. coli* (ATCC 33456) [38]. Moreover, *C. aksoyi* extract has also exhibited significant antibacterial activity against *E. coli*, with an inhibition zone of 7.00 ± 0.00 mm, and *C. amaena* against various Gram-positive and Gram-negative bacterial strains, with inhibition zones ranging from 7.00 to 11.00 mm [39]. In contrast, extracts from a different *Centaurea* species–*C. ainetensis* have demonstrated antibacterial activity with 88.8% susceptibility to the tested microorganisms [40].

The inter-study discrepancies in antimicrobial activity reported for *Centaurea* extracts may be explained by differences in extraction procedures, particularly the choice of solvent, which strongly influences both extraction yield and the spectrum of phytochemicals obtained. Previous phytochemical analyses of *Centaurea* species have identified a wide range of biologically active constituents, such as sesquiterpene lactones, flavonoids, phenolic acids and polyacetylenes, many of which have been recognized for their antimicrobial potential [41,42], and their presence may be strongly influenced by the extraction protocol.

The antimicrobial properties of plant extracts are generally determined by their complex phytochemical composition and cannot necessarily be attributed to a single constituent. In the present study, the phenolic content of the selected ethanolic extract was found to be dominated by apigenin derivatives. These are known to have a range of antibacterial effects, which influence bacterial membrane integrity, permeability, DNA gyrase activity and biofilm formation [43,44]. While this high abundance of apigenin derivatives may account for the observed antimicrobial activity, it should be noted that the study only evaluated the complete extract and not its isolated components or defined phenolic combinations; consequently, it is not possible to confirm any direct causal relationship between individual apigenin derivatives and the observed MIC or MBC values. Indeed, the extract may also contain a range of other constituents, including quercetin derivatives, caffeoylquinic acids, anthocyanins and hydroxycinnamoyl polyamines, which may also contribute to its overall antimicrobial effect. Further analysis based on bioactivity-guided fractionation and testing of isolated constituents is recommended to determine their individual and combined contributions.

Taken together, the biological assays indicate that the selected phenolic-rich ethanolic extract combines substantial antioxidant capacity and moderate antimicrobial activity, with preserved viability of L929 and Hs68 fibroblasts at concentrations up to 5 mg/mL. While these findings support an association between the complex phenolic composition of the extract and its biological properties, they do not conclusively establish which individual constituents are responsible for the observed effects. Moreover, the fibroblast assay provides evidence of in vitro cytocompatibility within the tested concentration range rather than comprehensive evidence of safety. Further studies involving bioactivity-guided fractionation, isolated compounds, mechanistic assays and more complex skin-related experimental models would be required to determine the compounds responsible for the observed activities.

## 3. Materials and Methods

### 3.1. Materials and Reagents

*Centaurea cyanus* flowers were obtained from a commercial supplier (ECOSPA S.C., Warsaw, Poland). Methanol, ethanol and chloroform were purchased from Avantor Performance Materials Poland S.A., Gliwice. Apigenin, axillarin, 3-caffeoylquinic acid, cyanidin-glicoside, kemferol 3-*O*-glucoside and 3-*O*-galactoside, and quercetin 3-*O*-glucoside and galactoside were purchased from Sigma-Aldrich (Steinheim, Germany).

### 3.2. Preparation of Cornflower Extracts

#### 3.2.1. Methanol Extract (1)

9.6 g of dried, ground cornflower flowers were poured over 80% methanol (approx. 300 mL) acidified with concentrated hydrochloric acid (1 mL of concentrated HCl per 1 L of methanol). The mixture was heated for one hour at the solvent’s boiling point under reflux. The extracted flowers were then filtered under pressure, and the resulting filtrate was concentrated to dryness on a rotary evaporator. The product was freeze-dried. The yield was found to be 10.2%, with 0.98 g of dry extract obtained.

#### 3.2.2. Methanol Extract (2) After Initial Defatting with Chloroform

Dried cornflower flowers (10.2 g) were poured with chloroform and left for 24 h. The next day, vacuum filtration was performed, and the flowers were left to dry overnight. The dried material (9.8 g) was ground and poured over 80% methanol (approx. 300 mL) acidified with concentrated hydrochloric acid (1 mL of concentrated HCl per 1 L of methanol). The mixture was heated for one hour at the solvent’s boiling point under reflux. The extracted flowers were then filtered under pressure, and the resulting filtrate was concentrated to dryness on a rotary evaporator. The product was freeze-dried. The yield was 10.1%, with 1.03 g of dry extract obtained.

#### 3.2.3. Ethanol Extract (3)

10.0 g of dried, ground cornflower flowers were poured over 70% ethanol (approx. 300 mL). The mixture was heated for one hour at the solvent’s boiling point under reflux. The extracted flowers were then filtered under pressure, and the resulting filtrate was concentrated to dryness on a rotary evaporator. The product was freeze-dried. 1.41 g of dry extract was obtained (yield 14.1%).

#### 3.2.4. Ethanol Extract (4) After Initial Defatting with Chloroform

Dried cornflower flowers (10.5 g) were poured with chloroform and left for 24 h. The next day, vacuum filtration was performed, and the flowers were left to dry overnight. The dried material (9.8 g) was ground and poured over 70% ethanol (approx. 300 mL). The mixture was heated for one hour at the solvent’s boiling point under reflux. The extracted flowers were then filtered under pressure, and the resulting filtrate was concentrated to dryness on a rotary evaporator. The product was freeze-dried. The yield was 13.7%, with 1.44 g of dry extract obtained.

#### 3.2.5. Water Extract (5)

10.3 g of dried, ground cornflower flowers were poured over distilled water (approx. 300 mL) acidified with concentrated hydrochloric acid (1 mL of concentrated HCl per 1 L of water). The mixture was heated for one hour at the solvent’s boiling point under reflux. The extracted flowers were then filtered under pressure, and the resulting filtrate was concentrated to dryness on a rotary evaporator. The product was freeze-dried. The yield was 5.9%, with 0.61 g of dry extract obtained.

#### 3.2.6. Water Extract (6) After Initial Defatting with Chloroform

Dried cornflower flowers (10.9 g) were poured with chloroform and left for 24 h. The next day, vacuum filtration was performed, and the flowers were left to dry overnight. The dried material (10.45 g) was ground and poured over distilled water (approx. 300 mL) acidified with concentrated hydrochloric acid (1 mL of concentrated HCl per 1 L of water). The mixture was heated for one hour at the solvent’s boiling point under reflux. The extracted flowers were then filtered under pressure, and the resulting filtrate was concentrated to dryness on a rotary evaporator. The product was freeze-dried. The yield was 6.0%, with 0.65 g of dry extract obtained.

### 3.3. UPLC/DAD/qTOF-MS/MS Analysis of Centaurea cyanus L. Extracts

Extracts (1–6) with corresponding masses: 199.7 mg, 204.2 mg, 203.8 mg, 206.7 mg, 205.4 mg, and 199.7 mg were dissolved in 1.5 mL of 80% methanol with 1% HCl.

10 µL of prepared extracts’ (1–6) solutions were applied sequentially to a column containing two solutions as the mobile phase: A: 0.1% formic acid in water, B: 0.1% formic acid in acetonitrile. Compound contents were expressed as mg/kg dry weight of plant material (mg/kg DM).

Polyphenolic compounds were characterized using an ACQUITY UPLC system (Waters Corp., Milford, MA, USA) equipped with a photodiode array detector and a G2 Q-Tof mass spectrometer (Waters, Manchester, UK) operating with an electrospray ionization (ESI) source in negative ion mode. Chromatographic separation was achieved on a BEH C18 column (1.7 μm, 2.1 × 100 mm; Waters) maintained at 30 °C. A 10 μL aliquot of each sample was injected, and analytes were eluted over a 15 min run using a linear gradient at a flow rate of 0.42 mL min^−1^, according to Chandran et al. [45]. The mobile phase consisted of solvent A (0.1% formic acid, *v*/*v*) and solvent B (acetonitrile).

Mass spectrometric analysis was conducted in full-scan mode across *m*/*z* 100–1500. Leucine enkephalin (500 pg mL^−1^, [M–H]^−^ = 554.2615 Da) was infused via the LockSpray system as an external reference. Instrumental settings were optimized as follows: capillary voltage, 2.5 kV; cone voltage, 30 V; source temperature, 100 °C; desolvation temperature, 300 °C; and nitrogen desolvation gas flow, 300 L h^−1^. Collision-induced dissociation was performed with argon at energies ranging from 0.3 to 2 V.

Data acquisition and processing were performed using MassLynx 4.0/ChromaLynx software (Waters). PDA spectra were collected from 200 to 600 nm at 2 nm intervals. Compounds were identified by comparing retention times and accurate mass measurements with the values for corresponding standards. When authentic reference standards were not available, the remaining compounds were tentatively identified based on their accurate mass, MS/MS fragmentation patterns, and chromatographic characteristics, supported by comparison with existing data.

### 3.4. Antioxidant Capacity

Antioxidant activity assessments were conducted using a Synergy H1 microplate reader (BioTek, Winooski, VT, USA) according to Chandran et al. [45]. The antioxidant potential of the *Centaurea cyanus* ethanolic extract (**4**) was determined using ferric reducing antioxidant power (FRAP), 2,2-diphenyl-1-picrylhydrazyl radical scavenging (DPPH), and 2,2′-azinobis(3-ethylbenzothiazoline-6-sulfonic acid) radical cation decolorization (ABTS).

For the FRAP assay, 10 µL of each sample was combined with 200 µL of freshly prepared FRAP reagent, vortexed for 30 s, and allowed to react for 10 min at room temperature before measuring the absorbance at 593 nm. In the DPPH assay, 10 µL of the sample was mixed with 200 µL of DPPH solution, incubated for 10 min, and the absorbance was recorded at 517 nm. For the ABTS assay, 10 µL of the sample was added to 200 µL of ABTS•^+^ solution, mixed thoroughly, and measured at 734 nm after a 10 min incubation period. A calibration curve was generated using Trolox (0–500 µmol/L) as the reference standard. Results are reported as micromoles of Trolox equivalents (TE) per 1 Gramm of extract (µmol TE/g).

### 3.5. Cytotoxicity Assay

The in vitro cytocompatibility of *Centaurea cyanus* ethanolic extract (4) was evaluated using reference mouse L929 fibroblasts (LGC Standards, Middlesex, UK) and human Hs68 skin fibroblasts. L929 cells were cultured in Roswell Park Memorial Institute (RPMI)-1640 medium, whereas Hs68 cells were maintained in high-glucose DMEM medium; both media were supplemented with 10% heat-inactivated fetal bovine serum (FBS), penicillin (100 U/mL) and streptomycin (100 µg/mL). Cells were maintained at 37 °C in a humidified atmosphere containing 5% CO_2_. Cell viability was evaluated according to ISO 10993-5 (Biological evaluation of medical devices—Part 5: Tests for in vitro cytotoxicity) using the MTT reduction assay as previously described [46]. Cells were used at a density of 2 × 10^5^ cells/mL and were exposed to the extract at concentrations of 0.5, 1, 2.5, 5 and 10 mg/mL. Cell cultures maintained in complete medium without extract served as the positive viability control (PC), whereas cells treated with 0.03% H_2_O_2_ served as the negative control (NC). Absorbance was measured at 570 nm using a Multiskan EX plate reader (Thermo Scientific, Waltham, MA, USA). Cell viability was calculated as follows: cell viability (%) = (absorbance of extract-treated cells/absorbance of untreated control cells) × 100. In accordance with ISO 10993-5, viability below 70% of the untreated control was considered indicative of an in vitro cytotoxic effect. Four independent experiments were performed, each in triplicate.

### 3.6. Antimicrobial Activity Assay

The antimicrobial activity of *Centaurea cyanus* ethanolic extract (4) was determined using the following reference microorganisms from the American Type Culture Collection (ATCC, Manassas, VA, USA): *Pseudomonas aeruginosa* ATCC 27853, *Escherichia coli* ATCC 25922, *Proteus mirabilis* ATCC 29906, *Staphylococcus epidermidis* ATCC 12228, *Staphylococcus aureus* ATCC 29213, *Bacillus cereus* ATCC 10876, *Cutibacterium acnes* ATCC 6919, *Candida albicans* ATCC 10231, *Candida glabrata* ATCC 2001 and *Candida parapsilosis* ATCC 22019.

We determined minimum inhibitory concentration (MIC), minimum bactericidal concentration (MBC), and minimum fungicidal concentration (MFC) values by broth microdilution according to European Committee on Antimicrobial Susceptibility Testing (EUCAST) recommendations and our previously described procedure [46]. We prepared and standardized fresh microbial suspensions before inoculation. The turbidity of the microbial suspensions was adjusted to a 0.5 McFarland standard prior to susceptibility testing. Mueller–Hinton broth was used for bacterial susceptibility testing, whereas RPMI-1640 medium was used for *Candida* spp. assays, following the laboratory approach used in our previous studies. We prepared serial dilutions of the extract in the appropriate growth medium and inoculated them with standardized microbial suspensions. Aerobic bacterial cultures were incubated for 24 h, while *Cutibacterium acnes* was incubated under anaerobic conditions for 48 h. *Candida* spp. were incubated for 48 h. MIC was defined as the lowest extract concentration showing no visible microbial growth. To determine MBC or MFC, aliquots from wells without visible growth were subcultured onto the appropriate agar medium; MBC/MFC was defined as the lowest concentration yielding no growth after subculture. Gentamicin and amoxicillin were used as antibacterial reference compounds, and amphotericin B as the antifungal reference compound. Growth and sterility controls were included in each experiment. Each assay was performed in three independent experiments.

### 3.7. Statistical Analyses

Statistical analyses were performed using Statistica 13 PL software (StatSoft, Kraków, Poland), and graphs were prepared using GraphPad Prism 10.0 (GraphPad Software Inc., San Diego, CA, USA). For the fibroblast cytocompatibility experiments, data from four independent experiments, each performed in triplicate, are presented as mean ± standard deviation (SD). The data distribution was determined using the Shapiro–Wilk test. Differences between untreated control cells and cells exposed to each concentration of the *Centaurea cyanus* extract were evaluated using the non-parametric Mann–Whitney U-test; separate comparisons were made for the L929 and Hs68 cell lines. Differences were considered statistically significant at *p* < 0.05. Antioxidant capacity data are presented as mean ± SD from four independent determinations. MIC, MBC and MFC values represent reproducible endpoints obtained in three independent antimicrobial experiments and were not subjected to inferential statistical analysis.

## 4. Conclusions

Among the tested *Centaurea cyanus* L. flower extracts, the highest phenolic content was achieved by defatting followed by ethanol extraction. This variant also exhibited substantial antioxidant capacity and moderate antimicrobial activity, particularly against *Escherichia coli*. It was also found to be cytocompatible with the tested L929 and Hs68 fibroblast lines. The present results demonstrate biological activity of the complete extract but do not establish direct causal relationships between individual phenolic constituents and the observed effects. However, further bioactivity-guided fractionation, mechanistic studies and additional functional assays, including skin-relevant models, are needed to identify the compounds responsible for these activities and to assess the potential application of *Centaurea cyanus* extracts in dermatological and cosmetic formulations.

## Figures and Tables

**Table 1:** UPLC-DAD ESI-MS detection and identification of phenolic compounds in extracts of *Centaurea cyanus* L. flowers.

Peak No.	R_t_	[M-H]^−^ (*m*/*z*)	MS/MS (*m*/*z*)	Compound	Methanol (1)	(CHCl_3_) Methanol (2)	Ethanol (3)	(CHCl_3_) Ethanol (4)	Water (5)	(CHCl_3_) Water (6)
1.	0.59	191	173	Quinic acid	141.45	146.71	275.50	415.02	100.00	119.16
2.	0.85	449	287	Eriodictyol hexoside	22.21	25.06	23.44	54.14	14.61	13.45
3.	1.65	341	191	Caffeoylhexose isomer 1	28.67	14.02	15.90	0.00	10.45	9.48
4.	1.79	341	179	Caffeoylhexose isomer 2	61.58	44.66	37.95	0.00	49.80	37.05
5.	2.80	355	193	Ferulic acid hexoside	2.27	1.97	1.24	47.07	0.00	0.95
6.	3.13	627	465/303/285	Taxifolin dihexoside isomer 1	57.56	57.67	93.34	113.41	0.00	37.12
7.	3.27	627	465/303/285	Taxifolin dihexoside isomer 2	53.60	53.12	67.05	73.45	29.19	35.48
8.	4.06	353	191	3-Caffeoylquinic acid *	2795.26	2801.96	3855.51	**4340.94**	1557.19	1447.89
9.	4.19	353	191	cis-3-Caffeoylquinic acid	392.75	397.45	185.39	254.60	246.08	256.35
10.	4.26	325	163	*p*-Coumaric acid hexoside isomer 1	863.03	867.61	1244.61	1500.71	405.69	445.21
11.	4.47	447	285	Kaempferol-3-O-galactoside *	195.50	198.59	281.62	259.65	126.26	125.46
12.	4.59	325	163	*p*-Coumaric acid hexoside isomer 2	146.10	148.02	222.75	204.43	50.73	60.34
13.	5.01	337	191	*p*-Coumaroylquinic acid isomer 1	446.54	449.24	650.25	785.12	264.82	246.36
14.	5.28	447	285.	Kaempferol 3-O-glucoside *	98.20	99.75	146.95	172.60	52.03	49.19
15.	5.57	607	431/269	**Apigenin-*O*-hexoside-*O*-glucuronide**	6123.52	7163.61	10,611.76	**13,632.80**	4912.14	4190.02
16.	5.64	693	445/269	Apigenin-*O*-glucuronide-*O*-(malonylhexoside)	60.12	53.42	87.80	109.16	42.16	42.22
17.	5.70	507	345/301	**Axillarin hexoside**	990.35	1011.53	1476.80	**1842.15**	719.13	635.44
18.	5.88	337	191	*p*-Coumaroylquinic acid isomer 2	60.07	61.16	95.88	90.42	41.67	38.13
19.	6.29	463	301	Quercetin galactoside *	217.07	218.22	301.46	266.99	130.45	117.77
20.	6.44	609	301	Quercetin hexoside rhamnoside	104.35	114.22	111.63	131.08	48.87	50.86
21.	6.84	463	301	Quercetin glucoside *	2338.82	2385.92	2623.52	**3163.95**	817.50	819.24
22.	7.26	505	301	Quercetin-3-*O*-(6″-acetyl)glucoside	371.65	401.83	832.62	961.91	95.74	107.58
23.	7.55	447	285	Kaempferol-3-*O*-hexoside	609.55	444.82	722.78	1063.00	141.26	188.33
24.	7.92	641	431/269	**Apigenin-3-*O*-glucoside-(6″-acetyl)glucoside**	3038.17	3021.27	4643.28	**5343.17**	1031.79	1050.54
25.	8.14	489	285	Kaempferol-O-acetylhexoside	157.18	227.75	333.21	357.87	35.89	49.69
26.	9.18	473	269	Apigenin-*O*-acetylhexoside	611.90	615.68	919.04	1119.83	178.07	192.89
27.	9.70	491	329/315	Isorhamnetin glucuronide	20.65	21.17	34.50	0.00	1.68	0.00
28.	10.14	395	233	Acetyl-caffeoyl quinic acid	16.35	38.18	48.67	81.39	0.00	0.00
29.	10.82	582	462/342/145/119	N^1^,N^5^,N^10^-tri-*p*-(*E*,*E*,*E*)-coumaroyl spermidine	296.02	980.25	991.10	2276.53	47.67	30.96
30.	10.99	269		Apigenin *	795.51	922.32	1585.05	**2110.79**	42.19	53.59
		**[M+H]^+^ (*m*/*z*)**	**MS/MS** **(*m*/*z*)**							
31.	3.18	611	449/287	Cyanidin-3,5-*O*-diglucoside	148.16	131.62	269.98	325.14	133.44	126.46
32.	3.94	697	536/449/287	Cyanidin-3-*O*-(6″-malonylglucoside)-5-*O*-glucoside	87.22	75.54	139.36	171.30	63.20	62.72
33.	4.38	711	549/449/287	Cyanidin-3-*O*-(6″-succinylglucoside)-5-*O*-glucoside	555.88	520.16	736.38	837.76	495.46	501.02
34.	4.64	625	287	Cyanidin-3-glucoside monoglucuronide	28.52	23.54	42.20	39.40	12.76	12.52
35.	5.17	535	287	Cyanidin-3-*O*-(6-*O*-malonylglucoside)	17.12	12.84	30.02	37.64	5.36	6.08
				**SUM [mg/kg DW]**	**21,952.90**	**23,750.88**	**33,738.54**	**42,183.42**	**11,903.28**	**11,159.55**
				**SUM [mg/g DW]**	**21.95**	**23.75**	**33.74**	**42.18**	**11.90**	**11.16**

* Compounds identified using corresponding authentic standards.

**Table 2:** Antioxidant capacity [µmol Trolox/g] of *Centaurea cyanus* L. ethanolic extract (4).

	DPPH [µmol Trolox/g]	ABTS [µmol Trolox/g]	FRAP [µmol Trolox/g]
M	240.34	170.53	189.93
SD	0.43	6.15	2.58

M—mean value; SD—standard deviation; n = 4.

**Table 3:** Antimicrobial activity of *Centaurea cyanus* extract, shown as minimal inhibitory concentration (MIC) and minimal bactericidal concentration (MBC) or minimal fungicidal concentration (MFC).

Microorganism	MIC/MBC/MFC (mg/mL)		MIC = MBC/MFC (mg/mL)		
			Gentamicin	Amphotericin B	Amoxicillin
	MIC	MBC/MFC			
Gram-negative bacteria					
*Pseudomonas aeruginosa* ATCC 27853	1	>10	<0.008	-	-
*Escherichia coli* ATCC 25922	0.5	1	<0.004	-	-
*Proteus mirabilis* ATCC 29906	1	>10	-	-	<0.001
Gram-positive bacteria					
*Staphylococcus epidermidis* ATCC 12228	1	2.5	<0.26	-	-
*Staphylococcus aureus* ATCC 29213	5	10	<0.002	-	-
*Bacillus cereus* ATCC 10876	5	10	<0.002	-	-
*Cutibacterium acnes* ATCC 6919	5	10	<0.002	-	-
Fungi					
*Candida albicans* ATCC 10231	2.5	>10	-	<0.001	-
*Candida glabrata* ATCC 2001	2.5	>10	-	<0.001	-
*Candida parapsilosis* ATCC 22019	2.5	>10	-	<0.001	-

Gentamicin, amoxicillin, and amphotericin B, broad-spectrum antibiotics, are used as antibacterial and antifungal reference substances; (-) were not tested.

## Data Availability

The original contributions presented in the study are included in the article; further inquiries can be directed to the corresponding author.
